# Peer Review of “Technologies to Support Assessment of Movement During Video Consultations: Exploratory Study”

**DOI:** 10.2196/32262

**Published:** 2021-09-24

**Authors:** Ebrahim Sadeghi-Demneh

**Affiliations:** 1 Musculoskeletal Research Center Isfahan University of Medical Sciences Isfahan Iran

**Keywords:** tele-rehabilitation, video-consultations, assessment of movement, eHealth, technology, desktop robots, wide-angle webcams, physical health, rehabilitation, remote, assessment, assistive technology, evaluation, framework, webcam, telehealth, robots

*This is a peer-review report submitted for the paper “Technologies to Support Assessment of Movement During Video Consultations: Exploratory Study.”*

## Round 1 Review

### General Comments

Thank you for taking the time to submit this paper [1]. It is an interesting area for health care practitioners. This was an exploratory trial on the feasibility of video consultation with some off-the-shelf technologies in the United Kingdom. This manuscript is well structured and written, but the external validity of the results is limited. I have included some feedback on the different sections of the manuscript and hope the authors will find these comments helpful.

### Specific Comments

#### Major Comments

1. Please consider that movement at least has four basic parameters, including force, range of motion/distance, rate (velocity/acceleration), and endurance (repeats until the mover is fatigued). I think authors could talk more about the shortcomings of their methods for comprehensive assessments of the parametric abilities of movements.

2. To further discuss the limitations of your study, please note that in resource-limited environments and developing countries, these results cannot be generalized.

#### Minor Comments

3. Please correct the spelling of “CINHAL”.

4. Please explain why authors selected a time limit (since 2016) for their literature search.

5. The specification of products/instruments should include details (model, manufacturer company, country).
